# The complementary role of lymphovascular invasion and perineural invasion in the TNM staging process of rectal cancer

**DOI:** 10.1097/MD.0000000000030687

**Published:** 2022-09-30

**Authors:** Tong Chen, Mingchuan Wang, Xianbin Cheng, Yizhuo Wang, Yang Jiang, Xuedong Fang, Huijie Xiao

**Affiliations:** a Department of Gastrointestinal, Colorectal, and Anal Surgery, China-Japan Union Hospital of Jilin University, Changchun, China; b Department of Thyroid Surgery, The Second Hospital of Jilin University, Changchun, China; c Department of Cancer Center, First Hospital of Jilin University, Changchun, China.

**Keywords:** high-risk factors, lymphovascular invasion, overall survival, perineural invasion, rectal cancer

## Abstract

The aim of this study is to clarify the association between lymphovascular invasion (LVI) and/or perineural invasion (PNI) and the clinical characteristics and prognostic importance of rectal cancer, to provide a basis for early adjuvant treatment of rectal cancer. We retrospectively analyzed patients diagnosed with rectal cancer. This study involved rectal cancer tissue samples were obtained by surgical methods. Data on histological form, tumor classification, tumor size, gross growth pattern, blood and lymphatic vessel invasion, and PNI of the slice by HE staining were obtained from pathological examination. Immunohistochemical analysis of tissue samples was performed to determine p53 and EGFR expressions. There were 330 rectal cancer patients included in the study. LVI and/or PNI can be used as a high-risk factor for the prognosis of rectal cancer, predict prognostic survival, and guide adjuvant therapy. The detection rates of LVI and PNI were 32.1% and 16.1%. Differentiation grade, Union for International Cancer Control staging, tumor-lymph node-metastasis staging are significantly related to LVI or PNI. Multivariate logistic regression analysis shows that poor differentiation and N ≥ 1 can be used as independent risk factors and predictive factors for LVI. At the same time, poor differentiation and T > 3 is an independent risk factor for PNI. Only poor differentiation is the risk factor for poor prognosis in Cox risk regression analysis. In addition, the simultaneous occurrence of LVI and PNI is an independent prognostic factor.

## 1. Introduction

Colorectal cancer is one of the world’s prevalent malignant tumors, and its morbidity and mortality rank third and fifth of all malignant tumors, respectively,^[1]^ and rectal cancer is 75% to 80% of colorectal cancer. The tumor-lymph node-metastasis (TNM) staging method of the Union for International Cancer Control (UICC) is now the most important determinant of postoperative adjuvant therapy for cancer of the colon and rectum. Lymphovascular invasion (LVI) and perineural invasion (PNI) are the essential aspect of solid tumor invasion and metastasis. Tumors may use blood vessels, lymphatics, and nerves via their microenvironment, as the “bridge” for invasion and metastasis is that the tumor develops collateral circulation with surrounding tissues. Therefore, tumor infiltration of the surrounding vascular nerves is a critical prerequisite for modifying the surrounding microenvironment in order to stimulate tumor growth and induce distant tumor metastasis and recurrence. The shed tumor cells metastasize far away via collateral circulation.^[2]^ On the other side, PNI can also occur in the absence of lymphatic or vascular invasions; tumor cells will secrete nerve growth factor and nerve growth factor receptor TrkA to modify the peripheral nerves and facilitate nerve penetration and metastasis via the nerve growth factor-nerve growth factor receptor TrkA signaling pathway.^[3]^ With the development of surgical technology and tumor molecular science, most patients with rectal cancer can be controlled or even cured to some extent after radical surgical resection and radiotherapy and chemotherapy. The diagnosis and treatment of rectal cancer is often dominated by TNM staging, which will direct adjuvant radiotherapy and chemotherapy before and after surgery to determine the prognosis. Just depending on TNM staging, though, has restrictions. In the ongoing analysis of tumors, individuals are increasingly understanding the biological actions of blood and lymphatic vessel infiltration and nerve infiltration, which complements not only the conventional TNM staging assessment but also is beneficial for rectal cancer assessment, this classification is incomplete since many patients at the same stage can have different outcomes. Opinions are different and divisive on the prognosis. At present, LVI or PNI have been independently researched by researchers. There are relatively few reports on the presence of nerve infiltration and vascular infiltration simultaneously. A significant number of patients had nerve penetration as well as blood and lymphatic vessel invasion in clinical practice. Therefore, in our center, we performed retrospective study and analysis on rectal cancer patients, using nerve and vascular invasion as hypotheses, investigating the association between the 2 with clinical features and recovery, and defining risk factors causing the poor prognosis of rectal cancer patients.

## 2. Patients and Methods

### 2.1 Patients

In accordance with the inclusion and exclusion criteria, 330 rectal cancer patients from the Department of Gastrointestinal Surgery, China-Japan Union Hospital of Jilin University from January 2014 to June 2015 were retrospectively included in the study. Patients with tumor recurrence, tumor stage IV, familial adenomatous polyposis, hereditary non-polyposis rectal cancer, and palliative surgery or local resection were excluded. None of the patients enrolled in the group received chemotherapy or radiotherapy. The surgical methods included laparoscopic surgery and open surgery. This study involved human tissue samples and was approved by the medical ethics committee of the China-Japan Union Hospital of Jilin University. The study of excised specimens obtained the patient’s informed consent. All patients with late clinical stage, poor pathological classification, and vascular and nerve invasion and other high-risk factors received conventional fluorouracil and oxaliplatin first-line chemotherapy after surgery.

When stratifying continuous variables, we refer to clinical guidelines and make judgments based on previous clinical experience: considering the survival rate of patients and the reduction of tolerance to treatment, 65 years of age is used as the stratification standard for patient age; considering the increased tumor malignancy and risk of metastasis, 5 cm was used as the stratification standard for tumor diameter.

### 2.2. Determination of LVI and PNI status

According to the American Joint Commission on Cancer/International Union Against Cancer (AJCC/UICC) staging method for TNM, 2 or more experienced pathologists reexamined the excised specimens of the primary tumor and the histological form, tumor classification, tumor size, gross growth pattern, blood and lymphatic vessel invasion, and PNI of the slice by HE staining were established. Vascular invasion is defined as tumor cells in the muscular layer of blood vessels or invading the muscular vascular endothelium, lymphatic vessel invasion is defined as the presence of tumor cell nests in the lymphatic cavity of non-muscular endothelial cells,^[4]^ and PNI is defined as the presence of tumor cells in the 3 layers of the nerve sheath, or, in close proximity to the nerve, affecting at least 33% of its entire circumference.^[5–7]^

### 2.3. Expression of EGFR and P53

We performed immunohistochemical studies for p53 and EGFR protein using an autoimmunostainer (Maixin, Fuzhou, China) in accordance with the manufacturer’s instructions. When more than 10% of the tumor cells were stained, the tumor was considered as positive for p53 and EGFR.

### 2.4 Statistical analysis

SPSS23.0 statistical software was used for data analysis, the measurement data were compared by rank sum test; the count data were compared by chi-square test or Fisher’s exact probability method for correction, and the difference was statistically significant with *P* < .05. In order to screen the final predictors of vascular nerve invasion, all candidate predictors with *P* < .05 in the univariate analysis were included in the multivariate logistic regression model. In the multivariate analysis, variables with *P* < .05 were considered as independent predictors. The Kaplan–Meier method was used to calculate the 5-year overall survival rate (OS), and the log-ranch test was used to evaluate the difference in survival rate. OS was measured from the date of diagnosis to death or the last follow-up visit. All *P* values are two-sided, and <.05 indicates statistical significance.

## 3. Results

In order to analyze the relationship between vascular and nerve invasion and the clinicopathological characteristics of rectal cancer, we analyzed the clinicopathological characteristics of 330 rectal cancer patients.

### 3.1. Patient and tumor characteristics

Among the 330 patients, 61.5% were males, most of them were young and middle-aged (67.9%) under 65 years of age. In terms of tumor pathology, UICC stages, II and III accounted for half of them, and most of them were highly or moderately differentiated adenocarcinoma (86.7%), mucinous adenocarcinoma accounted for only 17%, tumors with a diameter of less than 5 cm accounted for 63%, tumors invaded more than 71.2% below the muscularis propria, tumors without lymph node metastasis slightly more than tumors with lymph node metastasis (50.3% vs 49.7%), PNI is less common than LVI (16.1%vs32.1%). Tumor grade (*P* < .001), tumor stage (*P* < .001), T stage (*P* < .001), N stage (*P* < .001) is statistically different between colorectal cancer with and without LVI, which is not statistically significant with gender (*P* = .276), age (*P* = .616), mucinous adenocarcinoma (*P* = .755), tumor size (*P* = .393), p53 (*P* = .158), and EGFR (*P* = .786). PNI is statistically different in the tumor grade (*P* < .001), T stage (*P* < .001), N stage (*P* < .001), p53 (*P* = .032), which is not significantly different from gender (*P* = .123), age (*P* = .750), mucous glands cancer (*P* = .427), tumor size (*P* = .351), and EGFR (*P* = .227) (Table 1). Therefore, all candidate predictors with statistical significance (*P* < .05) in the univariate analysis were included as independent variables into the multivariate logistic regression model: the independent variables included in the regression analysis with LVI as the dependent variable were tumor grade, tumor stage, T stage, N stage; the independent variables included in the regression analysis with PNI as the dependent variable were tumor grade, tumor stage, T stage, N stage, and p53. According to logistic regression analysis, poorly differentiated tumor (*P* = .002) and lymph node metastasis (*P* = .002) are independent risk factors for LVI positive, and poorly differentiated (*P* = .018) and depth of invasion (*P* = .035) can predict PNI (Table 2).

**Table 1:** Clinicopathologic characteristics of the patients.

Clinical feature	LVI (+)	LVI (−)	*P* value	PNI (+)	PNI (−)	*P* value
Total	106	224		53	277	
Gender			.276			.123
Male	70	133		38	165	
Female	36	91		15	112	
Age (yr)			.616			.750
≤65	70	154		35	189	
>65	36	70		18	88	
Mucinous cancer			.755			.427
Yes	19	37		11	45	
No	87	187		42	232	
Tumor diameter			.393			.351
≤5	63	145		30	178	
>5	43	79		23	99	
Tumor grade			.000			.000
Poorly	26	18		16	28	
Moderately + well	80	206		37	249	
Tumor stage			.000			.000
I	4	69		0	73	
I	9	83		9	83	
III	93	72		44	121	
T stage			.000			.000
<3	12	83		1	94	
≥3	94	141		52	183	
N stage			.000			.000
<1	13	153		9	157	
≥1	93	71		44	120	
P53			.158			.032
Positive	77	179		35	221	
Negative	29	45		18	56	
EGFR			.786			.227
Positive	28	55		17	66	
Negative	78	169		36	211	

LVI = lymphovascular invasion, PNI = perineural invasion.

**Table 2:** Multivariate analysis of factors predicting colorectal cancer with LVI and PNI.

	LVI			PNI		
	OR	95% CI	*P*	OR	95% CI	*P*
Tumor grade	0.282	0.125–0.639	.002	0.398	0.185–0.855	.018
Tumor stage	1.573	0.344–7.191	.559	0.291	0.009–9.504	.488
T stage	2.285	0.893–5.851	.085	10.202	1.184–87.914	.035
N stage	23.830	3.137–181.016	.002	1.196	0.033–43.420	.922
P53				2.018	0.983–4.145	.056

CI = confidence interval, LVI = lymphovascular invasion, OR = odds ratio, PNI = perineural invasion.

### 3.2. Prognostic value of LVI or PNI and factors affecting survival outcomes

**Figure 1.**
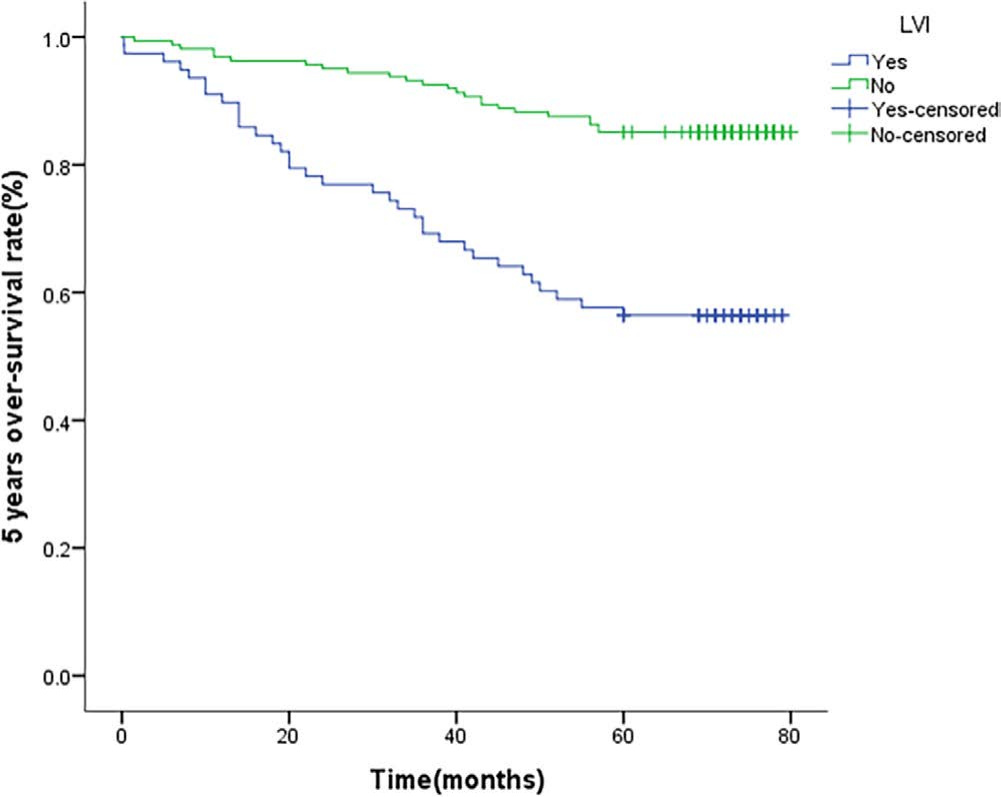
Unadjusted Kaplan–Meier survival analysis for lymphovascular invasion (LVI) related to overall survival in all patients.

**Figure 2.**
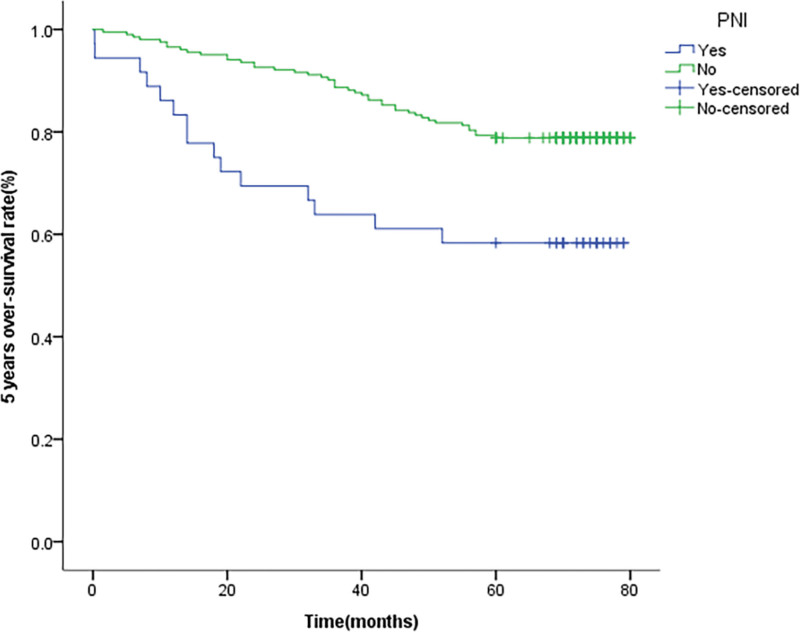
Unadjusted Kaplan–Meier survival analysis for perineural invasion (PNI) related to overall survival in all patients.

We conducted follow-up statistics on 330 patients, of which 91 patients were lost to follow-up and 239 patients received responses. The average follow-up time was 67.625 ± 1.481 months. The 5-year over-survival time for LVI or PNI was respectively 57.7% and 46.4%. We also performed Kaplan–Meier and Log-rank analysis to investigate the influence of clinicopathological factors on the prognosis of patients. It indicates that there is a substantial variation between LVI (*P* < .001) (Fig. 1), PNI (*P* = .002) (Fig. 2), and the OS. In addition, differentiation grade, pathological stage, depth of invasion T, and lymph node metastasis N are significant predictors of prognostic OS (Table 3). The multivariate model is then used to evaluate the effects of parameters found to have a significant impact on univariate analysis. Pathological evidence of LVI or PNI is not an independent predictor of OS (*P* = .117 vs .391), and only poorly differentiated tumor pathological types are independent prognostic factors for poor OS (Table 4).

**Table 3:** Univariate analyses of factors for 5-year overall survival (OS).

	Over survival		Log-rank	*P*
		95% CI		
Gender				
Male	67.030	62.886–71.174	0.052	.820
Female	68.695	64.581–72.810		
Age				
≤65	68.513	65.060–71.966	0.004	.952
>65	66.375	60.943–71.807		
Mucinous cancer				
Yes	62.830	55.497–70.162	0.940	.332
No	68.556	65.388–71.724		
Tumor grade				
Moderately + well	69.399	66.409–72.389	9.570	.002
Poorly	55.677	46.202–65.153		
Tumor diameter				
≤5	66.940	63.087–70.792	0.364	.546
>5	69.122	64.608–73.636		
Tumor stage				
I	76.018	72.081–79.954	32.596	.000
II	74.699	71.115–78.224		
III	59.804	54.972–64.636		
T stage				
<3	73.521	69.391–77.651	5.684	.017
≥3	65.327	61.578–69.077		
N stage				
<1	75.821	73.174–78.468	33.422	.000
≥1	59.634	54.773–64.496		
P53				
Positive	67.542	64.148–70.935	0	.988
Negative	68.533	62.655–74.411		
EGFR				
Positive	63.717	57.692–69.742	1.560	.212
Negative	69.089	65.791–72.387		
LVI				
Positive	56.301	50.069–62.533	26.562	.000
Negative	73.040	70.271–75.810		
PNI				
Positive	53.958	43.809–64.107	9.983	.002
Negative	70.106	67.278–72.934		
LVI and PNI				
Positive	46.803	35.028–58.578	22.823	0
Negative	70.481	67.748–73.214		

LVI = lymphovascular invasion, PNI = perineural invasion.

**Table 4:** Multivariate analyses of factors for 5-year overall survival (OS).

	OR	95% CI	*P*
Tumor grade	1.917	1.029–3.572	.040
Tumor stage	1.415	0.327–6.115	.642
T stage	1.343	0.546–3.301	.521
N stage	0.354	0.044–2.831	.328
LVI	1.658	0.044–2.831	.108
PNI	1.336	0.702–2.542	.377
LVI and PNI	0.494	0.261–0.936	.030

LVI = lymphovascular invasion, OR = odds ratio, PNI = perineural invasion.

### 3.3. The relationship between prognosis and simultaneous LVI and PNI

**Figure 3.**
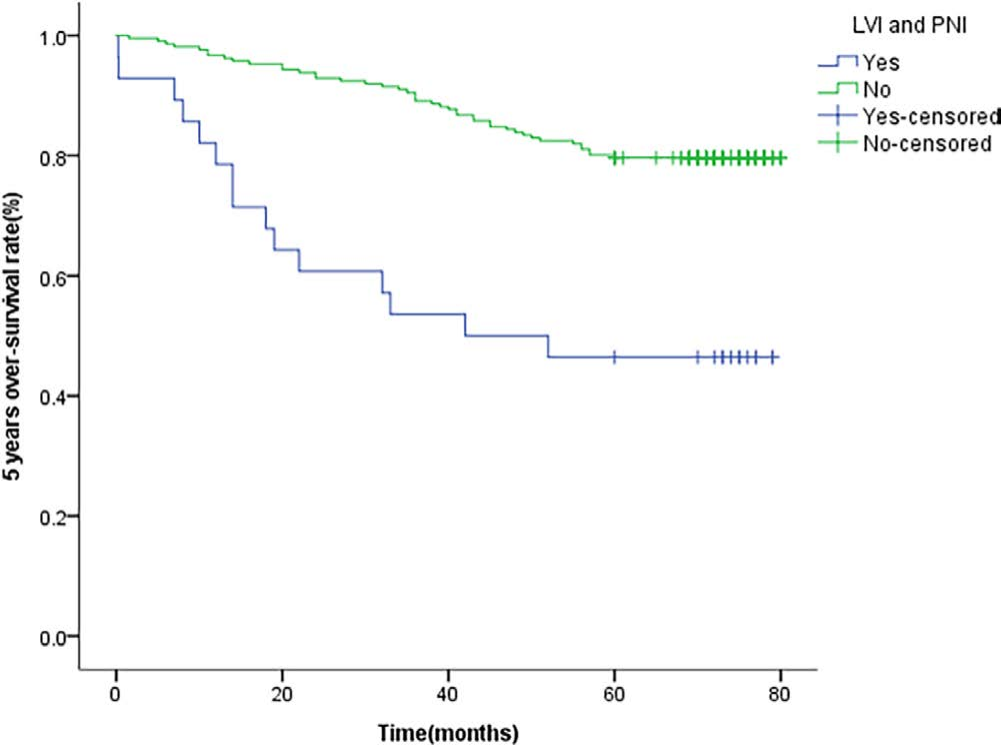
Unadjusted Kaplan–Meier survival analysis for lymphovascular invasion (LVI) and perineural invasion (PNI) related to overall survival in all patients.

In this study, because the kappa value of LVI and PNI was 0.416, the agreement was general. We analyzed the clinical characteristics of 43 patients with LVI and PNI at the same time. After multivariate analysis, poor tumor differentiation and T stage are independent risk factors. In addition, the simultaneous occurrence of LVI and PNI is significantly different from OS (Fig. 3) and is a prognostic predictor of OS (*P* = .030).

## 4. Discussion

In the study of tumor development, LVI and/or PNI has gained more and more interest in recent years. While the postoperative pathology study lacks uniformity, it has become the consensus to regard LVI and/or PNI as a significant outcome. The LVI and/or PNI happens predominantly at the stage of microcirculation of the tumor, which is the significant factor for early recurrence and malignant tumor metastasis,^[8]^ and may also increase the quality of staging and guide postoperative adjuvant chemotherapy. As high-risk factors such as T4 tumors, poorly differentiation, tumor perforation, blood and lymphatic vessel invasion and PNI, adjuvant chemotherapy are often recommended by specialists from the American Society for Clinical Oncology to be regularly used in patients with stage II colon cancer, which has also been verified by other scholars.^[9–11]^ However, related researches suggest that the impact of adjuvant therapy on patients with stage II colorectal cancer has little to do with the existence of high-risk factors.^[12,13]^

In our study, the frequency of LVI is 32.1%, and the spectrum of vascular invasion in colorectal cancer patients is between 8% and 89.5%. The definition of LVI and/or staining procedure may be the cause for such a significant transition. Generally, there are 3 types of vascular invasion: vascular penetration (submucosa and muscular layer) of the intestinal wall; outside the intestinal wall vascular penetration (periintestinal fat layer and fat layer subserosa); it invades the blood vessels inside and outside the wall.^[14]^ Not only LVI is a major indicator of postoperative recurrence of colorectal cancer, but it is also a prognostic factor for OS. Since the distribution of LVI is not clearly found in the mucosal layer, as the layers deepen, the rest of the submucosa, muscularis propria, and serosal layers are all distributed with abundant lymphatic and vascular networks. Therefore, BOSCH has conducted that 17 related studies on patients with early colorectal cancer also showed that LVI is one of the strongest independent predictors of lymph node metastasis,^[15]^ this is the same outcome as our study illustrates the connection between LVI and lymphatic metastasis. It remains contentious whether LVI will lead postoperative adjuvant therapy. Yasmeen and others found LVI to be a high-risk factor and claimed that the effects of adjuvant chemotherapy in patients with stage II were lower than in patients with stage III.^[16]^ Babaei et al observed that tumors with high-risk features (including blood and lymphatic vessel invasion) received enhanced survival benefits from adjuvant chemotherapy compared to low-risk tumors.^[17]^ Another research by Parnaby showed that LVI in patients with colorectal cancer was a significant factor in lowering total mortality and disease-free survival.^[18]^ Chand has indicated that both surgeons and oncologists agree that adjuvant chemotherapy in LVI-positive rectal tumors is a form of adjuvant chemotherapy that, after neoadjuvant chemoradiation, has been shown to help patients with LVI.^[19]^ Future studies should also concentrate on identifying biomarkers to predict LVI before surgery.

This study found that 16.3% of patients with rectal cancer have PNI, which is similar to previous studies, with an incidence of 9% to 30%, which is lower than that of blood and lymphatic vessel invasion. Via the molecular guidance of the nerve in the internal atmosphere along the concentration gradient,^[20]^ the malignant tumor itself will cause malignant tumor cells expand along the nerve channel, and this molecular feature is dependent on the existence of the tumor itself, that is, the pathological form and degree of differentiation. Huh has also shown that PNI varies between T3 and T4 in colorectal cancer^[5]^; Huang et al has shown that patients with differentiated tumors have a longer survival time and that patients with poorly differentiated tumors have a poor prognosis and a low degree of differentiation.^[21]^ As an independent risk factor for invasion of PNI, the degree of differentiation and depth of invasion of colorectal cancer is also linked to its invasion of the peripheral nerve, what is more, positive peripheral nerve invasion may occur in patients with poor differentiation and higher T stages. This outcome is consistent with the function of the tumor itself and the invasion of peripheral nerves. This research has also demonstrated that the degree of separation of the tumor is a manifestation of the degree of malignancy and a prognostic factor as well.

PNI guides postoperative adjuvant treatment and affects survival has been proven by extensive research. Quah’s study of 448 patients with stage II rectal cancer has demonstrated that PNI can be used to monitor postoperative adjuvant treatment as a high-risk factor.^[11]^ Zhou et al reported that PNI is an independent factor influencing the prognosis of colorectal cancer and used PNI as a TNM staging supplement.^[22]^ Patients with colorectal cancer II and III were split into 3 stages when paired with postoperative TNM staging and vascular invasion: stage II PNI negative, stage II PNI positive/stage III PNI negative, stage III PNI positive. It offers a basis for personalized adjuvant therapy in colorectal patients, but Chang claim that adjuvant treatment for patients with stage II colon cancer with high risk factors does not increase survival.^[23]^ Our study showed that PNI only single factor analysis was meaningful with survival, which is similar to Hu Gang study.^[24]^ The explanation may be that only we referred to metastases outside the nerve sheath, ignoring the invasion of the tumor within the PNI, and improving the false negative rate, creating mistakes. Therefore, full and unified diagnosis and treatment protocols for invasion of tumor nerves need to be established. In addition, we found that the positive expression of P53 is statistically different from that of PNI, and there is a strong correlation between the increase of p53 expression and PNI and poor differentiation in related reports.^[25]^

The depth of gastrointestinal tumor penetration, lymph node invasion, and remote metastasis have all been decisive factors for the prognosis of colorectal cancer patients, according to TNM staging; meanwhile, in early tumor metastasis, LVI and/or PNI is an significant occurrence and provides an important reference for early surgery or other adjuvant therapies. With the continuous improvement of endoscopic technology, endoscopic resection of neoplastic polyps is widely used. However, if LVI and/or PNI occurs in pathological reports, it is still controversial whether adjuvant treatment is required while further radical resection is considered. Further analysis of anti-tumor drugs produced for early LVI and/or PNI will have a beneficial effect on inhibiting early metastasis of the tumor and enhancing survival.

There were still some limitations in our study. Because this is a single center study, the number of patients included in this study is small. We hope that we can add more patients in the future to enrich our data.

## 5. Conclusion

In conclusion, LVI and/or PNI can be used as a high-risk factor for the prognosis of rectal cancer, predict prognostic survival, and guide adjuvant therapy.

## Author contributions

**Conceptualization:** Tong Chen, Huijie Xiao.

**Data curation:** Tong Chen, Huijie Xiao.

**Formal analysis:** Tong Chen, Huijie Xiao.

**Funding acquisition:** Huijie Xiao, Yizhuo Wang.

**Investigation:** Tong Chen, Mingchuan Wang, Huijie Xiao.

**Methodology:** Tong Chen, Mingchuan Wang, Huijie Xiao.

**Project administration:** Tong Chen, Huijie Xiao.

**Resources:** Tong Chen, Huijie Xiao.

**Software:** Tong Chen, Mingchuan Wang, Huijie Xiao.

**Supervision:** Xianbin Cheng, Yang Jiang, Huijie Xiao, Xuedong Fang.

**Validation:** Huijie Xiao.

**Visualization:** Huijie Xiao.

**Writing – original draft:** Tong Chen, Huijie Xiao.

**Writing – review & editing:** Tong Chen, Mingchuan Wang, Yizhuo Wang, Huijie Xiao.
